# Gene-activated matrix harboring a miR20a-expressing plasmid promotes rat cranial bone augmentation

**DOI:** 10.1093/rb/rbaa060

**Published:** 2021-03-13

**Authors:** Rena Shido, Yoshinori Sumita, Masahito Hara, Mayumi Iwatake, Shun Narahara, Mayumi Umebayashi, Kei-ichiro Miura, Yukinobu Kodama, Izumi Asahina

**Affiliations:** 1 Department of Regenerative Oral Surgery, Unit of Translational Medicine, Nagasaki University Graduate School of Biomedical Sciences, 1-7-1 Sakamoto, Nagasaki 852-8588, Japan; 2 Basic & Translational Research Center for Hard Tissue Disease, Unit of Translational Medicine, Nagasaki University Graduate School of Biomedical Sciences, 1-7-1 Sakamoto, Nagasaki 852-8588, Japan; 3 Laboratory of Craniofacial Tissue Engineering and Stem Cells, Faculty of Dentistry, McGill University, 3640 University Street, M43, Montreal, Quebec H3A 2B2, Canada; 4 Department of Hospital Pharmacy, Nagasaki University Hospital, 1-7-1 Sakamoto, Nagasaki 852-8501, Japan

**Keywords:** gene-activated matrix, in vivo gene transfer, bone augmentation, atelocollagen, mir20a

## Abstract

Gene-activated matrix (GAM) has a potential usefulness in bone engineering as an alternate strategy for the lasting release of osteogenic proteins but efficient methods to generate non-viral GAM remain to be established. In this study, we investigated whether an atelocollagen-based GAM containing naked-plasmid (*p*) DNAs encoding microRNA (miR) 20a, which may promote osteogenesis *in vivo* via multiple pathways associated with the osteogenic differentiation of mesenchymal stem/progenitor cells (MSCs), facilitates rat cranial bone augmentation. First, we confirmed the osteoblastic differentiation functions of generated *p*DNA encoding miR20a (*p*miR20a) *in vitro*, and its transfection regulated the expression of several of target genes, such as Bambi1 and PPARγ, in rat bone marrow MSCs and induced the increased expression of BMP4. Then, when GAMs fabricated by mixing 100 μl of 2% bovine atelocollagen, 20 mg β-TCP granules and 0.5 mg (3.3 μg/μl) AcGFP plasmid-vectors encoding miR20a were transplanted to rat cranial bone surface, the promoted vertical bone augmentation was clearly recognized up to 8 weeks after transplantation, as were upregulation of VEGFs and BMP4 expressions at the early stages of transplantation. Thus, GAM-based miR delivery may provide an alternative non-viral approach by improving transgene efficacy via a small sequence that can regulate the multiple pathways.

## Introduction

Bone engineering is a potential strategy to heal bone defects arising from trauma or disease, since it is considered less invasive, safer and of higher efficacy than traditional treatments such as autogenous bone grafts. For this reason, growth factors have been receiving the attention as key elements that can provide the osteo-inducibility to alloplastic substitutes on bone engineering [1, 2]. However, controlling their bioactivities at the transplanted site is unfortunately difficult due to several disadvantages, such as early inactivation with short half-lives and potential side effects [3, 4]. For instance, recombinant bone morphogenetic protein (rBMP) 2 or 4 has been shown to induce bone formation in various situations [1, 5, 6]. However, direct administration of high dose rBMPs is known to induce considerable swelling in patients, which may cause airway obstruction when applied to oral and cervical areas [4]. For such osteogenic growth factors, an efficient and controlled delivery system needs to be developed for bone engineering.

Bone engineering using gene-activated matrix (GAM), which is comprised of gene vectors encoding target proteins and a proper biodegradable matrix, has shown potential usefulness as an alternative strategy to achieve the sustained delivery of osteogenic proteins [7]. To facilitate the clinical application of this strategy, it needs to develop the effective conditions for *in vivo* gene transfer without the use of any cytotoxic transfection reagents or viral vectors [8–11]. Although viral vectors can efficiently transfer genes, there are considerable disadvantages such as the risk of virus-dependent recombination, immunogenicity or excessive protein expression exceeding the time period required for tissue recovery [12–14]. Therefore, non-viral plasmid-vectors have been frequently adopted for this strategy. However, there are still unsolved problems such as low efficiency of transfection to induce the bone regeneration [8–11]. For this reason, we have developed GAM composed of an atelocollagen and naked-plasmid (*p*) DNAs encoding osteogenic proteins such as BMP4 and have shown its possibility of simple and safe bone engineering [11]. Nevertheless, a significant dose (approximately 6 μg/μl) of *p*DNA was required to induce enough newly formed bone even when *p*BMP4 was incorporated. Therefore, non-viral GAMs display the high transgene efficacy and low toxicity should be further developed.

MicroRNAs (miRs) are non-coding RNAs which act as post-transcriptional regulators of multiple proteins and associated pathways. MiR17–92 cluster is highly conserved in all vertebrates, and members of this family play crucial functions in the development of tissues and organs [15]. Among these miRs, some have been identified to play pivotal functions during osteoblast proliferation, differentiation, mineralization, and extracellular matrix synthesis. Particularly, several miRs, such as miR15b, miR20a, miR26a, miR29b and miR30c, have been reported to positively regulate osteoblast differentiation by inhibiting the negative regulation of osteoblasts [16–20]. For instance, miR29b functions in the promotion of osteogenesis by both suppressing the inhibitors of cell signaling pathways required for osteogenesis, such as HDAC4, TGFβ3, ACR2A, CTNNBIP1 and DUSP2, and modulating the expression of collagen genes during extracellular matrix maturation in mouse MC3T3 cells [20]. Such miRs have recently been thought to be promising as novel therapeutic agents in bone tissue engineering because of several significant advantages, such as including a small sequence and having many genetically important target genes. Therefore, GAM-based miR delivery may provide an alternative non-viral approach by improving transgene efficacy via a small sequence that can regulate the multiple pathways associated with bone formation. However, there have been only limited studies on GAMs that apply microRNAs for bone regeneration.

Given this background, we investigated whether atelocollagen-based GAM containing naked-*p*DNAs encoding miR20a (*p*miR20a) could facilitate rat cranial bone augmentation, which is considered as a model of regenerative therapy on dental implant treatment for jawbone atrophy. MiR20a has recently been suggested to positively control the expressions of BMP2/4 and Runx2 in human mesenchymal stem cells (MSCs) during their osteoblastic differentiation via the inhibition of plural negative regulators that maintain the balance between osteoblast and adipocyte differentiation, such as Crim1 (an antagonist of BMPs), Bamb1 (an antagonist of BMP type I receptor) and PPARγ (negative regulator of BMP/Runx2 expressions) [17]. Furthermore, miR20a may function to induce angiogenic gene expression in stromal cells under hypoxic conditions [21]. As mentioned above, we previously confirmed that atelocollagen-based GAM can reliably induce engineered bone when *p*BMP4 or *p*Runx2 are incorporated at a high dose (more than 6 μg/μl) of *p*DNA [11]. Therefore, we hypothesized that GAM-based miR20a delivery may function efficiently using less *p*DNA because it includes a small sequence and can simultaneously control multiple pathways, including BMP/Runx2 signaling.

## Methods

### Plasmid preparation

All animal (rat) experiments were performed in accordance with the relevant ethical guidelines, and all procedures were approved by the Nagasaki University Animal Ethics and Use Committee (1812051492). The miR20a (sequence: UAAAGUGCUUAUAGUGCAGGUAG) overexpression vector, pCytomegalovirus (pCMV)-mir20a-internal ribosome entry site (IRES)-green fluorescent protein (GFP) (MI0000638) (SC403217), and its control vector, pCMV-mir-IRES-GFP (PCMVMIR), were purchased (Origene Technologies, Rockville, MD, USA).

### Isolation of mesenchymal stem cells from rat compact bone

F344 rats (five-week-old, male) (CLEA Japan Inc., Tokyo, Japan) were sacrificed, and the femur and tibia are harvested after removing the muscles. Then, the bones were broken into small pieces using bone scissors. After washing with phosphate buffered saline (PBS), the bone fragments were enzymatically dissociated in Dulbecco’s modified Eagle’s medium (DMEM, Sigma Aldrich) supplementing with 0.1% type II collagenase (Sigma Aldrich, St. Louis, MO, USA), 10% fetal bovine serum (FBS, Sigma Aldrich) and 2% antibiotics/antimycotics for 1 h at 37°C. The digests were filtered by a 70 µm cell strainer (Corning Falcon, NY, USA) and the bone fragments remaining on the cell strainer were washed three times with PBS. The bone fragments were cultured with DMEM containing 10% FBS and 2% antibiotic–antimycotic solution in 10 cm plates at 37°C under 5% CO_2_. After 3 days of culture, the bone fragments were removed, and the medium was changed. When cultured cells reached 80% confluence, they were sub-cultured as mesenchymal stem/progenitor cells (MSCs) until passage 3 (P3) for subsequent experiments.

### Transfection of miR20a into MSCs, and gene expression analysis of transfected cells

A total of 5 × 10^4^ P3 MSCs were seeded per well in 12 well plates. Then, the culture medium was replaced to the medium mixed with miR20a-pDNA-dendrigant-poly-L-lysine (DGL)-γ-polyglutamic acid (γPGA) complex vector (nanoball vector) (5 µg per well) after 24 h [22]. Regarding this vector, we previously found that a *p*DNA complex coated with biodegradable γ-PGA provided adequate gene expression without cytotoxicity [22, 23]. Also, the biodegradable DGL that has sterically congested cations has been shown to enhance the gene expression. Therefore, the ternary complexes (*p*DNA–DGL–γPGA complexes), which show to be stable nanoparticles, can exhibit markedly high gene expression and low cytotoxicity. In fact, the *p*DNA–DGL–γPGA complexes also had high gene expression in the marginal zone (rich dendritic cells) of spleen after intravenous injection into mice [23]. Furthermore, in the preliminary experiment of this study, we confirmed its high transfection efficacy without cytotoxicity for miR20a delivery to cultured MSCs when compared with the commercial vectors such as a lentiviral vector (data not shown).

At 2 h post-transfection, the transfection medium was removed and subsequently cultured for 7 days. At 24 h after transfection, transfected cells were observed for GFP expression under the confocal microscope (LSM 800 with Airyscan; Carl Zeiss, Inc., Oberkochen, Germany). Then, total RNA was extracted from the MSCs by employing the mirVana^TM^ miRNA isolation kit (Invitrogen, Waltham, MA, USA), and RNA was transcribed with the TaqMan MicroRNA Reverse Transcription Kit (Applied Biosystems, Foster City, CA, USA). miR20a expression was analyzed by the TaqMan universal master mix (Applied Biosystems) with U6 RNA as an internal control for normalization. Mx3000p real-time PCR system (Agilent Technologies, Santa Clara, CA, USA) was used for qRT-PCR. Subsequently, MSCs were harvested after 2 and 7 days post-transfection to assess the mRNA expressions that are regulated by miR20a such as *crim1*, *bambi1*, *pparγ* and *bmp4*. Total RNA was extracted by using TRI reagent (Molecular Research Center Inc., Cincinnati, OH, USA), and the ReverTra Ace qPCR RT Kit with gDNA Remover (TOYOBO, Osaka, Japan) was employed for cDNA synthesis. Quantitative polymerase chain reaction was performed using SYBR green and gene specific primers on a Mx3000p real-time PCR system. Table 1 shows the rat-specific primer sets, and glyceraldehyde-3-phosphate dehydrogenase (*gapdh*) was used as an internal standard.

**Table 1.** Rat primer sets

Gene	Forward primer	Reverse primer
*bambi*	5′-CAGCCTCTTGTTCTCACTTC-3′	5′-ACCTCATCACTAAGGTGCAG-3′
*crim1*	5′-AATGTGTGCAGCTGTCCATG-3′	5′-AGCTGCTACTCCGAGTCTTG-3′
*pparγ*	5′-GCTGTGAAGTTCAATGCACTGG-3′	5′-GTTCAGCTTCAGCTGGAGTTCC-3′
*bmp4*	5′-CACTGTGAGGAGTTTCCATCAC-3′	5′-AGGAGATCACCTCATTCTCTGG-3′
*vegf-a*	5′-TTGTTCAGAGCGGAGAAAGC-3′	5′-TTTAACTCAAGCTGCCTCGC-3′
*vegf-b*	5′-CTCATGATCCAGTACCGAGC-3′	5′-GCTTCACAGCACTCTCCTTTC-3′
*dusp2*	5′-CAGCACTGCAACACGAGATG-3′	5′-AGCTGTTATTTTCGGCCCCA-3′
*f4/80*	5′-ACCTGCCACAACACTCTTGG-3′	5′-TCACAAGATTCTTCCAGGCCC-3′
*cd206*	5′-TTCCTTTGGACAGACGGACG-3′	5′-TCCCTGCCTCTCGTGAATTG-3′
*gapdh*	5′-TGCACCACCAACTGCTTAG-3′	5′-GGATGCAGGGATGATGTTC-3′

### Preparation of gene-activated matrix

**Figure 1.**
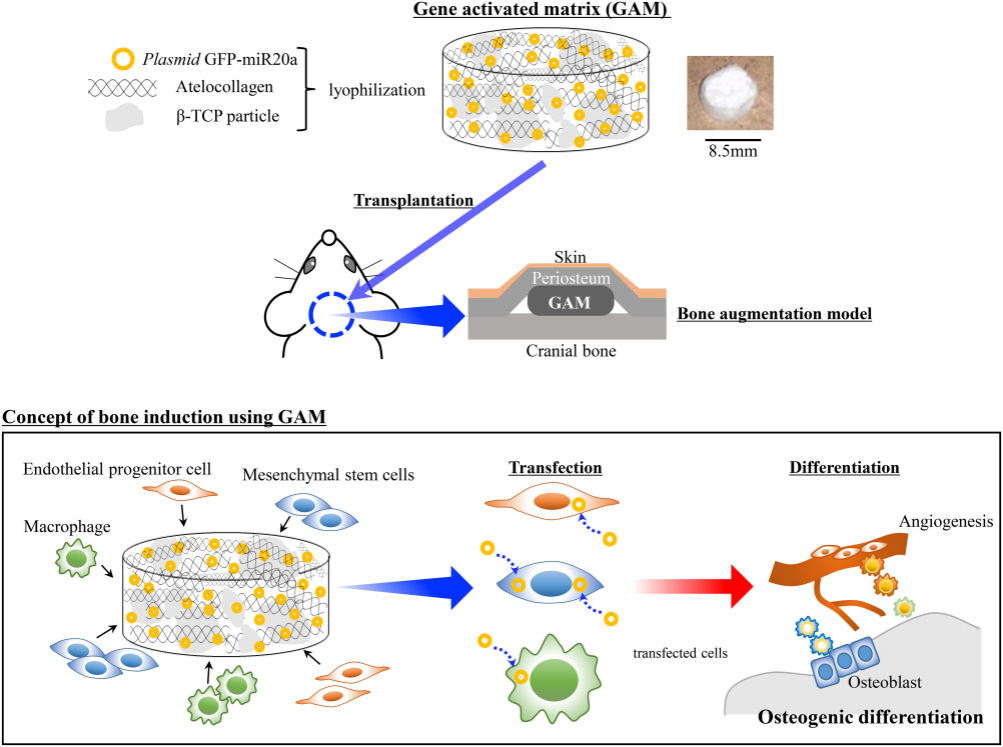
Schematic diagram describing the experimental design for the preparation and transplantation of GAMs for bone engineering

For all experiments, GAMs were prepared the day before transplantation. 0.5 mg of CMV–GFP plasmid-vectors harboring of miR20a were dissolved in 60 µl of sterile water, and mixed together with 20 mg of β-TCP granules (G1 Osferion; OLYMPUS, Tokyo, Japan) and 100 µl of 2% bovine atelocollagen (Atelocollagen Implant, KOKEN, Tokyo, Japan) at the lids of 1.5 ml Eppendorf tubes. These mixtures were lyophilized overnight (Fig. 1). The vehicle of CMV–GFP plasmid alone was applied as an experimental control for this study. All experiments were carried out in each experimental group, such as the *p*GFP group, transfected with only the *p*GFP vector, and the *p*miR20a group, transfected with *p*miR20a-GFP.

### GAM transplantation

Seven-week-old male rats (F344) were anesthetized by exposing them to 5% isoflurane gas (Wako, Osaka, Japan), and kept warm during and after the operation. GAMs were onlay-grafted onto the cranial bone surface under the periosteum as a vertical bone augmentation model (Fig. 1) [*n* = 96; 3 rats/group (each group of *p*GFP and *p*miR20a containing 0.25 or 0.5 mg *p*DNAs, respectively) at each time point (5 days, 1 and 2 weeks post-transplantation); 5 rats/group (each group of *p*GFP and *p*miR20a containing 0.25 or 0.5 mg *p*DNAs, respectively) at each time point (1, 4 and 8 weeks post-transplantation)]. At 1, 4, and 8 weeks, specimens were harvested to evaluate the transfection efficiency and histologically new bone formation. Furthermore, at 5 days and at 1 and 2 weeks post-transplantation, the specimens were harvested to determine specific gene and protein expressions. In addition, to ensure the efficacy of GAM harboring *p*miR20a, OCP/Col scaffolds (TOYOBO, Otsu, Japan) that were seeded 0.5 mg of *p*GFP and *p*miR20 were transplanted to the rat calvaria bone defects (5 mm of diameter) (3 rats/group). Then, the specimens were harvested at 4 and 8 weeks of transplantation and the new bone formation was analyzed by micro-CT and histological observations.

### Detection of transfected cells

At 1 week post-transplantation, the specimens were harvested for detecting the transfected cells. Harvested specimens were fixed with 4% paraformaldehyde and then decalcified with a solution containing 2.9% citric acid, 1.8% trisodium citrate dehydrate, 10% formic acid and 90% distilled water. After embedded in paraffin wax, five-micrometer sections were used for immunohistochemical staining with rabbit anti-GFP antibody (1:500) (Table 2). Then, the slides were incubated with anti-rabbit secondary-antibody (1:500) using a Vectastain ABC kit (Vector Laboratories, Burlingame, CA, USA). Finally, the specimens were reacted with 0.1% w/v 3,3′-diaminobenzidine tetrahydrochloride (DAB) (DAB immunohistochemistry; Gen Way Biotech, San Diego, CA, USA) and counterstained with hematoxylin. Negative staining was carried out by replacing the first antibody with pre-immune serum eluted from the corresponding affinity columns. Tissues were examined by a light microscope under 200× magnification.

**Table 2.** Antibodies for immunostaining and western blotting

Antibodies	Company, Catalog No.
Rabbit polyclonal anti-GFP antibody	Abcam, #6556
Rabbit polyclonal anti-PPARγ antibody	CST, #2435
Rabbit polyclonal anti-BMP4 antibody	Abcam, #39973
Mouse monoclonal anti-βactin antibody	Abcam,# 6276
Anti-rabbit IgG, HRP-linked antibody	CST,# 7074
Goat anti-mouse IgG HRP	Abcam, #97023

### mRNA and protein expressions in transplants

After 5 days and at 1 and 2 weeks post-transplantation, the specimens were harvested and pulverized using a homogenizer (MP-Biomedicals, Tokyo, Japan). Total RNAs and proteins were extracted by using TRI Reagent. Quantitative PCR was employed to detect the mRNA expressions of miR20a-related osteogenic (*crim1*, *bambi*, *pparγ*, *bmp4*), vasculogenic (vegfa, *vegfb*, *dusp2*) and macrophage (*f4/80*, *cd206*) genes in specimens. A ReverTra Ace qPCR RT Kit with gDNA Remover was used for cDNA synthesis. Then, the qPCR was performed using SYBR green and gene specific primers on a Mx3000p real-time PCR system. Table 1 shows the rat-specific primer sets, and glyceraldehyde-3-phosphate dehydrogenase (*gapdh*) was used as an internal standard.

Western blotting was performed using proteins extracted from specimen lysates. Protein lysates were separated by using Sodium dodecyl sulfate-Polyacrylamide gel electrophoresis (SDS-PAGE) (10%) and transferred to Poly Vinylidene Di-Fluoride (PVDF) membranes. Then, they were blocked with 5% non-fat dry milk for 1 h and incubated with anti-BMP4 and PPARγ antibodies overnight (Table 2). After rinsing, membranes were reacted with a Horse Radish Peroxidase (HRP)-conjugated secondary-antibody (Cell Signaling Technology, Danvers, MA, USA), and proteins detected by chemiluminescence. βactin was used as an internal loading control (Table 2).

### Histological observations

To assess the bone augmentation at 4 and 8 weeks after transplantation, the specimens were harvested and fixed with 4% paraformaldehyde. Then, they were decalcified by using a solution containing 2.9% citric acid, 1.8% trisodium citrate dehydrate, 10% formic acid, and 90% distilled water, and embedded in paraffin wax. After that, deparaffinized sections (5-μm thickness) were stained with hematoxylin and eosin (H&E). The volume of augmented bone-like tissues was analyzed by NIH ImageJ software (NIH, Bethesda, MD, USA), and the percentage of surface area occupied by bone-like tissues was observed by light microscopy under 40× magnification using five sections from each specimens per group (five specimens in a group). Two examiners measured the newly formed bone area after independently choosing sections randomly. Masson’s trichrome staining were performed on the slides at 4 and 8 weeks post-transplantation for 15 min at 56°C in Bouin’s solution (Sigma Aldrich), and then the excessive stain was removed from the slides by washing under running tap-water. The nuclei were stained with Weigert’s iron hematoxylin (Sigma Aldrich) for 5 min, and the slides were washed in tap-water for 5 min and rinsed with distilled water. The slides were left in a phosphotungstic/phosphomolybdic acid solution for 5 min and then Aniline Blue solution for 5 min. Finally, the slides were reacted with 1% acetic acid for 2 min and fixed by mounting medium (Muto Pure Chemicals, Tokyo, Japan). Five sections were employed for this staining from each of five specimens per group.

To label the new bones at 4 weeks post-transplantation by double calcein labeling assay, calcein (8 mg/kg, Wako) was intraperitoneally injected into the rats at the time of GAM transplantation and one day before sacrifice. After fixation and embedding in methyl methacrylate, specimens were cut into 30–40 µm thick sections and stained with toluidine blue. The double calcein labeling was captured with a fluorescence microscope under 200× magnification.

### Statistical analysis

The means were analyzed using one-way analysis of variance. Dunnett’s multiple-comparison *t*-test was applied to find the significant differences within each group. Experimental values were shown as means ± SD, a *P* value of <0.05 was considered statistically significant.

## Results

### Biological function of pmiR20a on cultured MSCs

All experiments in this study were carried out in each group, such as the *p*GFP group, transfected with only the *p*GFP vector (pCMV-mir-IRES-GFP) as a control group, and the *p*miR20a group, transfected with *p*miR20a-GFP (pCMV-mir20a-IRES-GFP) as an experimental group.

**Figure 2.**
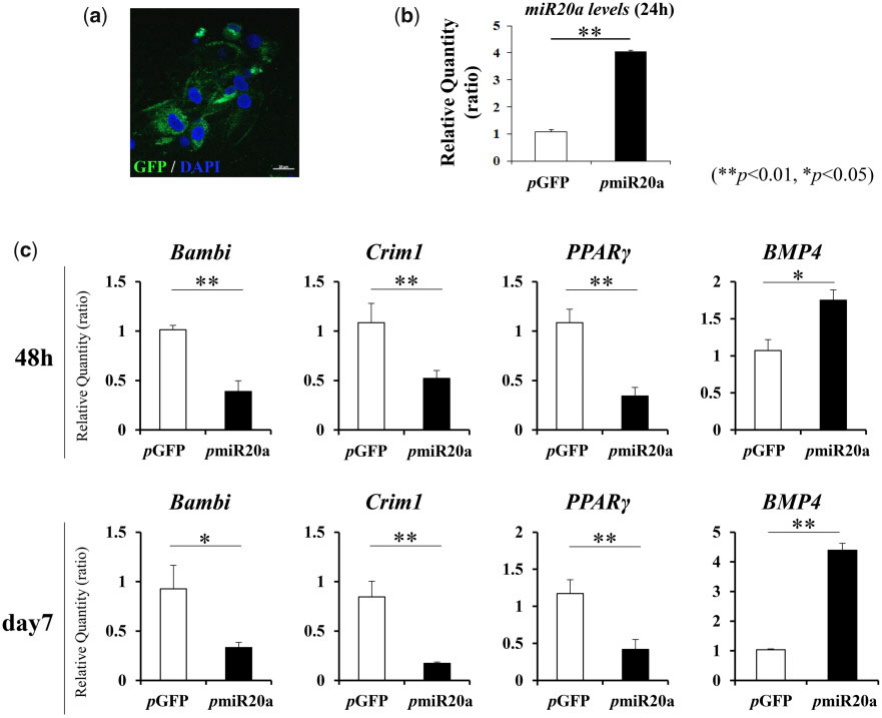
Biological activity of *p*miR20a in cultured MSCs after transfection. (**a**) GFP expressions in MSCs after 24 h post-transfection. Scale bar is 50 µm. (**b**) MiR20a expressions in MSCs at 24 h after transfection of *p*GFP or *p*miR20a (**P < 0.01, *P < 0.05). (**c**) Expression changes of co-regulator genes of the BMP signaling pathway (via miR20a) and the BMP4 gene (**P < 0.01, *P < 0.05)

First, the biological function of generated *p*miR20a on the osteoblastic differentiation of MSCs was analyzed *in vitro*. At 24 h post-transfection, GFP signals were detectable in cultured MSCs (Fig. 2a), and the expression of miR20a in MSCs after *p*miR20a transfection was significantly upregulated compared to that in MSCs after *p*GFP (without miR20a) transfection (Fig. 2b). Then, at 2 and especially 7 days of culture, we found that *p*miR20a transfection led to the inhibition of mRNA expressions of Crim1, Bambi1 and PPARγ, which are co-regulators of the BMP signaling pathway via miR20a in osteoblastic differentiation of MSCs (Fig. 2c). Related to this, BMP4 gene expression was markedly upregulated at these time points (Fig. 2c).

### Detection of transfected cells and gene expressions related to new bone formation in transplants

**Figure 3.**
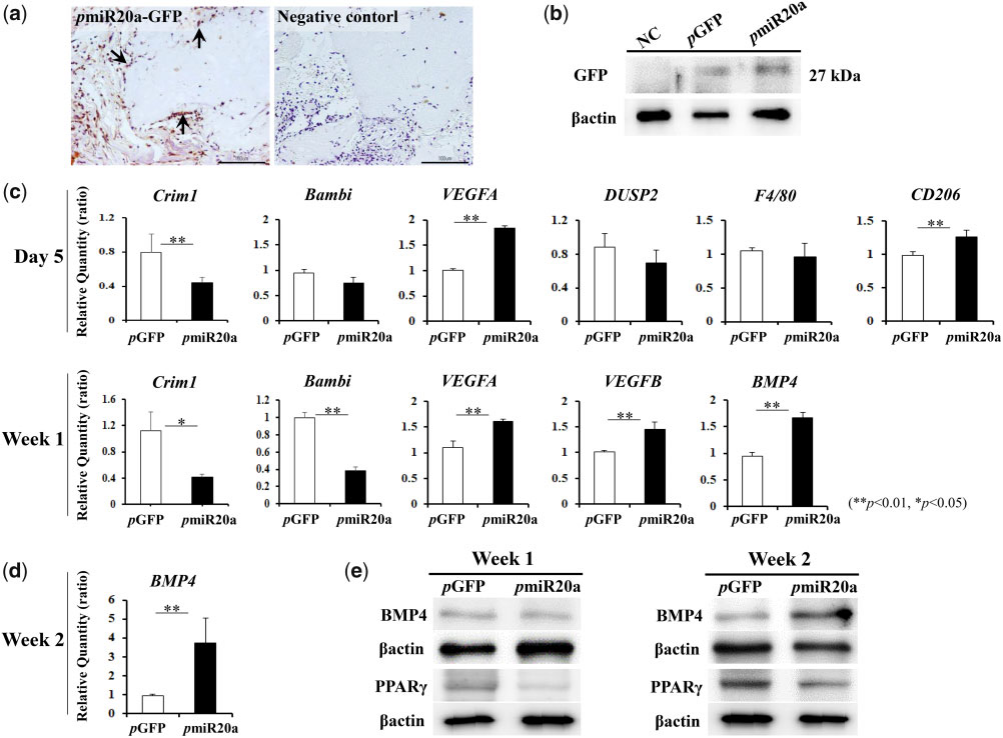
Detection of biological activity of *p*miR20a *in vivo* [number (*n*) of specimens; 3 rats/each group of *p*GFP and *p*miR20a at each time point (5-days, 1- and 2-weeks post-transplantation)].(**a**) GFP expression (arrow) of migrated cells at the surface area of β-TCP granules in *p*miR20a samples 1 week after transplantation. Scale bar is 50 µm. **(b)** Protein levels of GFP in *p*GFP and *p*miR20a samples at 1 week. (**c**) Expression levels of miR20a-related osteogenic, vasculogenic and macrophage genes at 5 days and 1 week post-transplantation (**P < 0.01, *P < 0.05). (**d**) BMP4 mRNA levels in transplants at 2 weeks (**P < 0.01). (**e**) Protein levels of BMP4 and PPARγ 1 and 2 weeks after transplantation.

When GFP expression in transplanted GAMs harboring 0.5 mg *p*miR20a was assessed at 1 week post-transplantation, GFP-positive cells were detected around the surface of β-TCP granules (Fig. 3a). Also, western blotting showed GFP expression in transplants of both 0.5 mg *p*GFP and *p*miR20a groups (Fig. 3b). At Day 5 and Week 1 post-transplantation, mRNA expressions of Crim1 and Bambi in specimens of the *p*miR20a group were decreased (Fig. 3c). In addition, at Week 1, BMP4 gene expression was upregulated in the *p*miR20a group (Fig. 3c), though there was no obvious change in its protein level between the *p*GFP and *p*miR20a groups (Fig. 3e, left). However, increased BMP4 expressions were clearly observed at both the mRNA and protein levels in transplants of GAMs harboring pmiR20a at Week 2 (Fig. 3d and e, right). Meanwhile, protein expressions of PPARγ in *p*miR20a specimens were decreased both at Weeks 1 and 2 (Fig. 3e). Furthermore, VEGFA and CD206 (MRC1; a marker of M2 macrophages) genes were upregulated in the *p*miR20a group after 5 days of transplantation, though there were no obvious changes in the expression of DUSP2 (a repressor of angiogenesis) and F4/80 (a marker of M1/M2 macrophages) mRNAs (Fig. 3c). These characteristics may indicate that *p*miR20a promotes angiogenic and anti-inflammatory phenotypes in transplants. VEGFA and VEGFB expressions were also increased in the *p*miR20a group 1 week after post-transplantation (Fig. 3c).

### Histological analysis of bone augmentation

**Figure 4.**
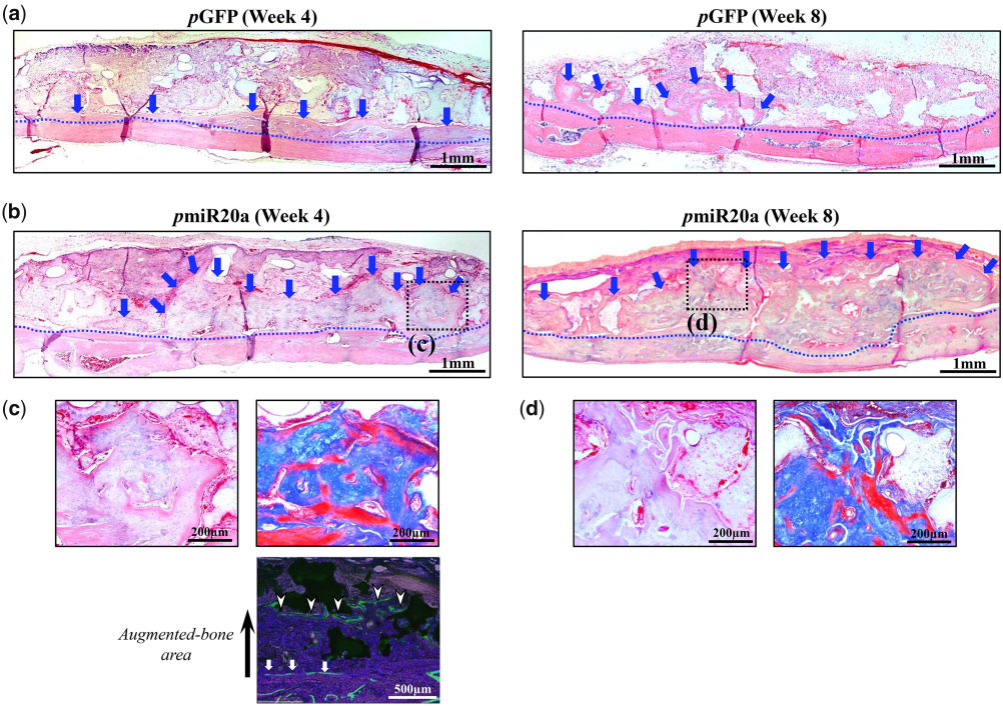
Histological appearance at 4 and 8 weeks after transplantation of GAM onto the rat cranium [number (*n*) of specimens; 5 rats/each group of *p*GFP and *p*miR20a at each time point (4- and 8-weeks post-transplantation)]. (**a, b**) HE staining of specimens in *p*GFP and *p*miR20a groups, respectively (representative images). Substantial bone formation was seen along the calvarial bone in the *p*miR20a group (b) when compared with the *p*GFP group (a). Scale bar; 1 mm, blue dotted line; boundary of the cranium and newly formed bone, blue arrow; augmented bone area. **(c, d)** The black box areas in (b) are shown in higher magnification. Augmented bone clearly surrounded β-TCP granules (left panels, HE; right panels, Masson’s trichrome staining). Scale bar is 200 µm. (c) Cacein double labeling of pmiR20a samples at 4 weeks. Scale bar is 500 µm.

**Figure 5.**
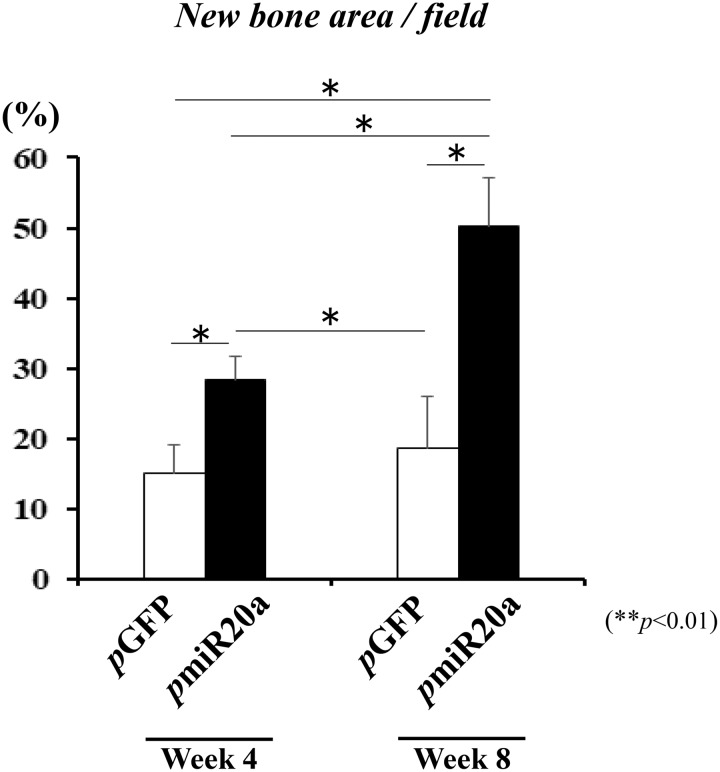
Bone formation area at 4 and 8 weeks post-transplantation (**P < 0.01) [number (*n*) of specimens; 5 rats/each group of *p*GFP and *p*miR20a at each time point (4- and 8-weeks post-transplantation)].

At 4 weeks following the transplantation of GAM to the cranium (as a vertical augmentation model), new bone formation was observed both in 0.5 mg *p*GFP and *p*miR20a groups (Fig. 4a and b). However, substantial bone formation was seen along the calvarial bone in samples of the *p*miR20a group, whereas limited amounts of bone formation were observed at the immediate proximity of calvarial bone in the *p*GFP group (Fig. 4a and b). Calcein double labeling showed the actual bone augmented area in the *p*miR20a group up to 4 weeks after transplantation (Fig. 4c), and at the surface of absorbed β-TCP granules, replacement new bone tissue, which had osteocytes, was recognized (Fig. 4c). This area was revealed to be immature bone tissue by Masson’s trichrome staining (Fig. 4c). At 8 weeks post-transplantation, we found that bone tissues were largely augmented when transplanted with GAMs harboring 0.5 mg *p*miR20a, while no obvious new bone formation was detectable in the *p*GFP specimens (Fig. 4a and b). The augmented bone seemed to be mature in the *p*miR20a samples because absorption of β-TCP granules surrounded by new bone had progressed further. However, at the far site from the host bone, the new bone tissues were still immature, though the replacement of bone tissues had progressed in the surface area of β-TCP granules (Fig. 4d). The amount of newly formed bone by GAMs harboring *p*miR20a at 4 weeks was 28.44 ± 3.25% in the entire area of the transplants, while that by *p*GFP was 15.00 ± 4.06% (Fig. 5). Subsequently, the amount of augmented bone in the *p*miR20a group at 8 weeks had reached 50.24 ± 6.79%, but that in the *p*GFP group had only reached 18.76 ± 7.34% (Fig. 5). In addition, to further confirm the regenerative capability of GAM composed of OCP/Col disc and *p*miR20a, we transplanted them to the calvarial bone defects with 5 mm diameter in rats. As results, the micro-CT and histological analyses were presented (see Supplementary Fig. S1a–d), and the new bone tissues were recognized ubiquitously in the *p*miR20 and *p*GFP groups at 8 weeks post-operatively, but this phenomenon seemed to be prominent in the *p*miR20 group.

## Discussion

This study verified the potential of direct miR delivery with alloplastic substitutes, which may provide a straightforward and effective strategy for bone augmentation. The positive outcomes of this study were (i) miR20a reliably induced BMP4 expression in rat cultured MSCs after transfection by inhibiting its multiple negative regulators, (ii) *p*miR20a also gradually promoted BMP4 expression in transplanted GAMs by inhibiting the negative regulators of BMP signaling without pro-inflammatory actions and (iii) resulting bone augmentation was promoted when atelocollagen-based GAM harboring *p*miR20a was transplanted to the rat cranial bone. These outcomes indicate that there are several advantages in applying miRs for GAM-based bone engineering.

The overexpression of miR20a in cultured rat MSCs led to the upregulation of BMP4 gene expression with the downregulation of negative regulator genes of BMP signaling such as Crim1 and Bambi (which are antagonists of BMP2/4/7 and their type I receptors, respectively), and PPARγ (which positively regulates adipogenesis and negatively regulates BMP/Runx2 expressions) [24–28]. This evaluation was based on the findings of a previous work using human cultured MSCs as shown by Zhang *et al.* [17]. They found that individual siRNAs of these negative regulators improved the osteogenic differentiation of MSCs via BMP signaling and demonstrated that these regulators are certainly targets of miR20a. Therefore, miR20a delivery must function effectively to promote BMP2/4 signaling and subsequent osteoblastic differentiation of MSCs. Indeed, our results are consistent with these previous findings and also demonstrate that such pathways of BMP4 can be regulated by miR20a delivery *in vivo*. Actually, after 1 week of transplantation, transfected cells were detectable around the surface of β-TCP granules in specimens of the *p*miR20a group. These cells should have differentiated into osteoblasts, because BMP4 production in GAMs was certainly increased 2 weeks after transplantation due to inhibition of its negative regulators (Crim1, Bambi, and PPARγ) starting at least 5 days post-transplantation. Subsequently, bone augmentation was obviously promoted up to 8 weeks post-transplantation in those specimens. Meanwhile, it has been shown that miR20a represses the p38 pathways via binding to the 3′UTR region of MKK3 mRNA, which represses endothelial migration and then impairs the capillary formation in an angiogenic engineered model [29]. Contrary to this report, the expressions of VEGFs in specimens of the *p*miR20a group were markedly upregulated from the early stages of transplantation. Related to the angiogenic effects of miR20a, Lin *et al.* [21] reported that miR20a activates the Extracellular Signal regulated kinase (ERK) pathway via direct repression of the DUSP2 gene and then promotes angiogenic gene expression in hypoxic stromal cells. However, in this study, there were no obvious changes in DUSP2 expression in GAMs harboring *p*miR20a after transplantation. Therefore, we assume that increased production of BMP4 in MSCs might induce VEGFs expression in MSCs or endothelial progenitor cells (EPCs), and that this step may be critical to the process of neovascularization in transplanted GAMs. In fact, the secretion of VEGFs by MSCs has been suggested to enhance the endothelial differentiation of EPCs via a paracrine mechanism [30]. Moreover, interestingly, upregulation of CD206 mRNA expression was observed in GAM specimens harboring *p*miR20a at 5 days post-transplantation without apparent changes in F4/80 gene expression. The detailed reasons for this phenomenon are unknown, but this result suggests that the delivery of miR20a has the effect of enhancing the M2 profile of macrophages. miR20a is known to negatively regulate pro-inflammatory cytokines release in rheumatoid fibroblast-like synoviocytes by directly targeting ASK1 expression [31]. Moreover, a recent investigation has shown that supplementation of BMP2 leads to the diminished expression of the M1 phenotype in M1 polarized macrophages and promotes macrophage differentiation into osteo-specific phenotypes via the secretion of osteogenic and angiogenic factors for bone healing [32]. At any rate, delivery of miR20a to osteogenic sites must function to coordinate diverse osteogenic and angiogenic factors in a temporospatial fashion during osteogenesis.

With respect to actual osteoinduction *in vivo*, we found that GAM harboring *p*miR20a at a concentration of 3.3 μg/μl could markedly promote bone augmentation on the rat cranium. The areas occupied by newly formed bone tissue in *p*miR20a group specimens were increased by approximately 1.9-fold and 3-fold at 4 and 8 weeks, respectively compared to those of the *p*GFP specimens. This reliably demonstrated that naked *p*mirR20a can be delivered with GAM comprised of atelocollagen and β-TCP granules and induce vertical bone augmentation due to high transgene efficacy. Previously, we showed that naked *p*BMP4 can induce bone augmentation when incorporated into atelocollagen-based GAM containing more than 6 μg/μl of *p*DNA [11]. Therefore, regulating the multiple pathways associated with bone formation by miR may be highly advantageous for GAM-based (cell-free) bone engineering. MiRs are expected to become successful agents for gene therapy or tissue engineering because of several considerable advantages, including their small size sequences that are often conserved among species completely [19, 33]. However, few studies have shown the usefulness of non-viral GAMs using miRs to date. In the present study, we employed naked *p*miR because we considered *in vivo* delivery of a small amount of naked *p*DNAs or *p*miRs with only a biodegradable scaffold to be a simple and low-toxic method. Indeed, administration of *p*DNAs is judged to be safe *in vivo*, particularly when applied at a low concentration. Even large amounts of naked *p*DNAs, such as 1–16 mg doses, have been transplanted to the local sites for treating cancer or limb ischemia. Their safety has been indicated by phase I/II clinical trials [34, 35]. We then focused on an atelocollagen matrix as a scaffold for GAM, because atelocollagen-mediated nucleic-acid delivery systems have greatly progressed for the treatment of various diseases [35]. In particular, researchers who developed the atelocollagen-based gene delivery have indicated that 5 μg/μl is the proper nucleic-acid concentration for local transplantation [36]. Therefore, such GAMs may be able to deliver *p*miR20a safely at a low-dose concentration *in vivo*. In addition, atelocollagen-based GAM including β-TCP may provide an appropriate space in local sites for gradual release of genes. Meanwhile, a recent study has shown that polyplexes carrying miR26a promote bone repair effectively by enhancing both endogenous osteogenesis and angiogenesis when implanted with a nanofibrous 3D scaffold into mouse cranium bone defects [19]. Therefore, many challenges in establishing miR delivery can be overcome by including gene vectors and biodegradable matrices without adding cells.

In conclusion, GAM, comprised of atelocollagen and *p*miR20a, reliably promoted vertical bone augmentation in rats by high transgene efficacy via multiple pathways. The results in this study suggest that miRs are promising for GAM-based bone engineering using non-viral gene delivery systems.

## Supplementary data

Supplementary data are available at *REGBIO* online.

## Supplementary Material

rbaa060_Supplementary_Data
