# Supplementary material for: Gene-activated matrix harboring a miR20a-expressing plasmid promotes rat cranial bone augmentation
Source: Regen Biomater. 2021 Mar 13;8(2):rbaa060. doi: 10.1093/rb/rbaa060 (PMC7955717; doi:10.1093/rb/rbaa060)
Supplement: rbaa060_Supplementary_Data [file rbaa060_supplementary_data.zip › 2020.11.23 Revised Suuplementary Figure 1.docx]

**Supplementary Figure1.** μCT and histological appearances at 4 and 8 weeks after GAM transplantation of cranial bone defect [number (*n*) of specimens; 3 rats/each group of *p*GFP and *p*miR20a at each time point (4- and 8-weeks post-transplantation)]. Representative images of µCT in specimens of *p*GFP and *p*miR20a groups. **(a)** Regenerated bone tissues were found ubiquitously in the *p*GFP and *p*miR20 groups, but this phenomenon seemed to be prominent in the *p*miR20 group. **(b)** Considerable bone formation was recognized histologically in the specimens of GAM harboring *p*miR20 at 8 weeks. Scale bar; 5mm, yellow dotted line; boundary of the cranium and regenerated bone, black arrow; area of regenerated bone tissues. **(c, d)** Black box areas in **b** are shown in higher magnification. Regenerated bone clearly surrounded β-TCP granules (HE staining). Scale bar is 500 µm.
