# Supplementary figures and images for: Gene-activated matrix harboring a miR20a-expressing plasmid promotes rat cranial bone augmentation
Source: Regen Biomater. 2021 Mar 13;8(2):rbaa060. doi: 10.1093/rb/rbaa060 (PMC7955717; doi:10.1093/rb/rbaa060)

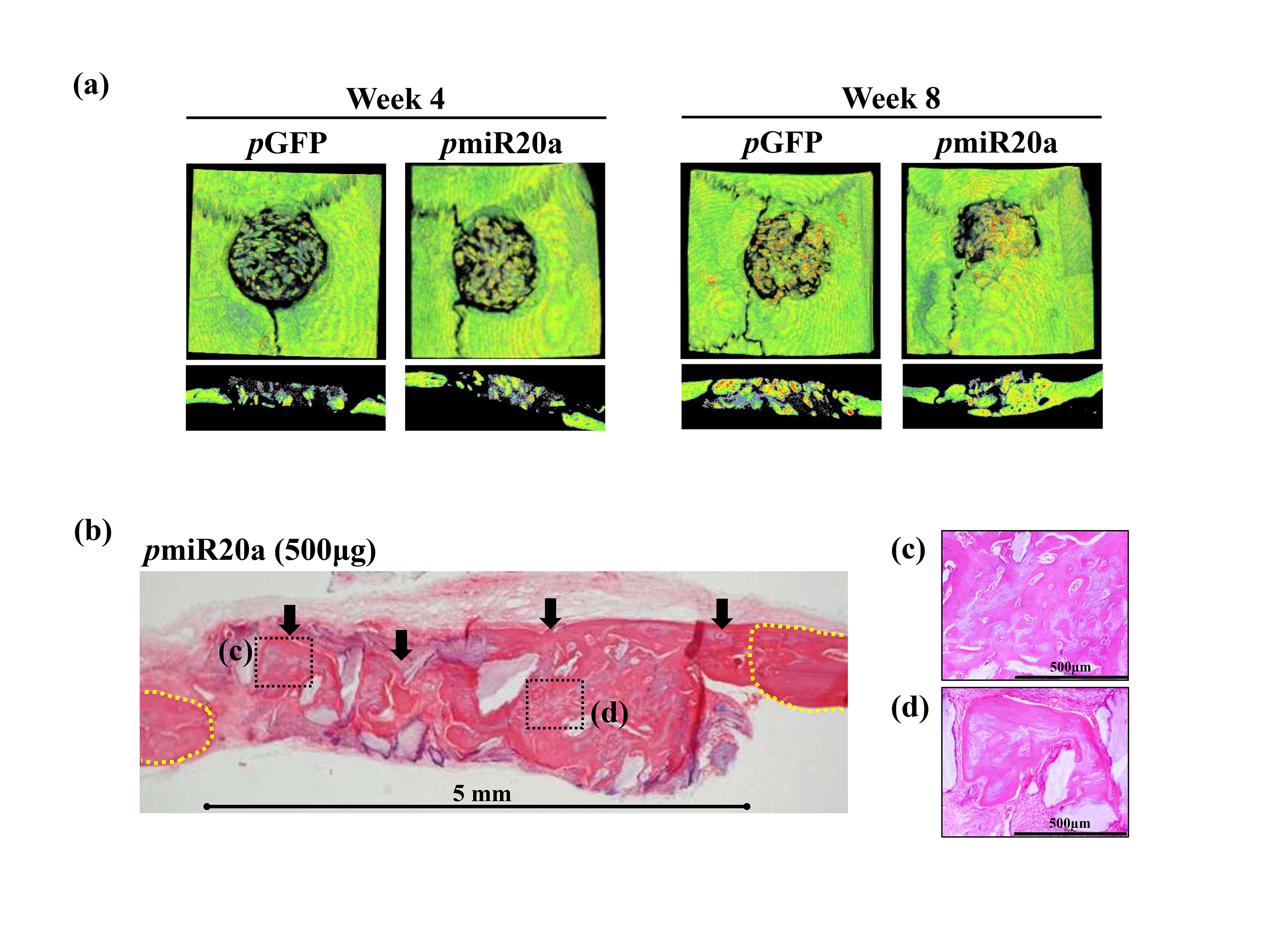

Supplement: rbaa060_Supplementary_Data [file rbaa060_supplementary_data.zip › Supplementary Figure 1 for Shido et al.tif]
